# Efficacy and safety of short-term spinal cord stimulation and pulsed radiofrequency in the treatment of postherpetic neuralgia: a meta-analysis

**DOI:** 10.3389/fneur.2025.1586995

**Published:** 2025-06-18

**Authors:** Zehao Liu, Yan Weng, Funing Liu, Dezhou Jiang, Chunmei Wu, Yong Chen, Xiaoxia Duan, Qing Zhong

**Affiliations:** ^1^Department of Anesthesiology, The Affiliated Hospital of Southwest Medical University, Luzhou, China; ^2^Department of Anesthesiology, The People's Hospital of Jianyang, Jianyang, China; ^3^Chengdu Medical College, Chengdu, China

**Keywords:** meta-analysis, pulsed radiofrequency, postherpetic neuralgia, short-term spinal cord stimulation, treatment

## Abstract

**Objective:**

This study aimed to explore the efficacy and safety of short-term Spinal Cord Stimulation (stSCS) and Pulsed Radiofrequency (PRF) in the treatment of postherpetic neuralgia (PHN).

**Methods:**

We searched the PubMed, Cochrane Library, Web of Science, Embase, China Biological Medicine Database (CBM-disk), Chinese National Knowledge Infrastructure (CNKI), Wanfang, and VIP databases for randomized controlled trials (RCTs) from the establishment of the database to August 1, 2024. Review Manager 5.4 and Stata 18.0 were used for the meta-analysis.

**Results:**

In total, eight randomized controlled trials comprising 479 patients were included. Meta-analysis showed that compared with PRF, stSCS had better pain relief (*p* < 0.01), lower Pain Rating Index Affective (PRI-A) score (*p* < 0.01), lower Pain Rating Index Sensory (PRI-S) score (*p* = 0.002), better sleep quality (*p* = 0.02), higher effective rate (*p* < 0.01), and lower incidence of postoperative complications (*p* = 0.007). However, complete remission rate (*p* = 0.24) after the two treatment methods were similar between the two groups. Moreover, stSCS treatment is more expensive.

**Conclusion:**

In general, stSCS is a more effective and safe method for the treatment of PHN, but its high cost is an unavoidable problem. Each method has advantages and disadvantages that should be considered comprehensively in clinical practice.

**Systematic review registration:**

https://www.crd.york.ac.uk/PROSPERO/, identifier CRD42024576536.

## 1 Introduction

Herpes zoster (HZ) is caused by the reactivation of the latent varicella-zoster virus in the cranial nerves or dorsal root ganglia. The virus spreads along the sensory nerves to the dermatomes (1–3). The primary manifestations comprise rash and radicular pain (3). Postherpetic neuralgia (PHN) is the most common complication of HZ and is usually defined as pain lasting more than 3 months (4). The nature of the pain often manifests as burning, stabbing, cutting, or electric shock and so on. Furthermore, based on persistent pain, it is often accompanied by severe hypersensitivity reaction (5). Pain usually affect the sleep and daily life of patients, leading to anorexia, weight loss, fatigue, depression, withdrawal from social activities and employment, and even loss of independent living ability (1). A study showed that the incidence rate of HZ in China is 4.28/1000 person-years, and for individuals aged ≥ 60 years, it is 11.69/1000 person-years. The risk of suffering from PHN is 12.6% (6). Because the pathogenesis of PHN is unclear, there is currently no treatment for this condition. Clinically, the therapeutic goals are to control pain as early and effectively as possible, relieve accompanying sleep and emotional disorders, and improve the quality of life. Conventional treatments include drug, physical, and interventional therapy (4, 7).

The mechanism of Spinal Cord Stimulation (SCS) is derived from the Gate Control Theory proposed by Melzack and Wall (8). The electrodes are placed in the epidural space of the spinal canal, and the spinal cord is stimulated by an electric current to block the transmission of pain signals to the brain to achieve pain control. In 1967, SCS was first reported to be used in the treatment of chronic pain (9). After decades of development, SCS has been approved by the U. S. Food and Drug Administration for the treatment of various chronic intractable pain conditions that affect the trunk or extremities, such as complex regional pain syndrome, failed back surgery syndrome, post-stroke pain, ischemic lower limb pain, painful diabetic neuropathy, and refractory non-surgical back pain (10). Previous studies have shown that SCS is also a reasonable choice for patients with PHN (11, 12). As a new mode of SCS, short-term SCS (stSCS) requires patients to be treated for 1–2 weeks. It has been widely used in the treatment of HZ-related pain due to its convenience and effectiveness (11, 13–15).

Pulsed Radiofrequency (PRF) is a minimally invasive treatment for chronic pain, and was proposed by Sluijter (16). PRF relieves neuropathic pain by inhibiting the release of excitatory neurotransmitters induced by pain (17). Animal studies have confirmed that PRF is a safe and effective treatment method for reducing neuralgia (18, 19), with minimal nerve damage (20, 21). Existing clinical studies have confirmed its role in refractory neuropathic pain such as PHN, cervical or lumbar radicular pain, failed back surgery syndrome, and various peripheral neuralgia (22–25). Therefore this technique has great potential and clinical application value in the treatment of chronic pain, particularly in patients with PHN (26–28).

Recently, Xue et al. conducted a meta-analysis on stSCS and PRF in the treatment of HZ-related pain and concluded that stSCS is superior to PRF in terms of analgesic effect and safety (29). However, as the development outcome of refractory HZ, the treatment effect of PHN is often different from that in the early stage (25). Currently, there is no definitive conclusion regarding the effectiveness, safety, and economic benefits of these two treatments in patients with PHN. Therefore, we conducted a meta-analysis to compare the advantages of these two methods in the treatment of PHN.

## 2 Methods

This study was conducted following the Preferred Reporting Items for Systematic Reviews and Meta-Analyses (PRISMA 2020) statement (30). This meta-analysis was registered in PROPERO under the registration number CRD42024576536^1^.

### 2.1 Search strategy

We searched the PubMed, Cochrane Library, Web of Science, Embase, China Biological Medicine Database (CBM-disk), Chinese National Knowledge Infrastructure (CNKI), Wanfang, and VIP databases. The search period was from the establishment of the databases to August 1, 2024. Through the Medical Subject Headings thesaurus, we identified the key words mainly including ‘Neuralgia, Postherpetic,’ ‘Spinal Cord Stimulation,’ and ‘Pulsed Radiofrequency Treatment.’ We concatenated all retrieved subject terms and free words using Boolean logic operators. The full search strategy for each English database is shown in the Table 1.

**Table 1:** The full search strategy for each database.

	Search strategy
PubMed	((((Neuralgia, Postherpetic[MeSH Terms]) OR (Neuralgia, Postherpetic[Title/Abstract])) OR (Postherpetic Neuralgia[Title/Abstract])) AND ((((Spinal Cord Stimulation[MeSH Terms]) OR (Spinal Cord Stimulation[Title/Abstract])) OR (Cord Stimulation, Spinal[Title/Abstract])) OR (Stimulation, Spinal Cord[Title/Abstract]))) AND ((((((((Pulsed Radiofrequency Treatment[MeSH Terms]) OR (Pulsed Radiofrequency Treatment[Title/Abstract])) OR (Pulsed Radiofrequency Treatments[Title/Abstract])) OR (Radiofrequency Treatment, Pulsed[Title/Abstract])) OR (Radiofrequency Treatments, Pulsed[Title/Abstract])) OR (Treatment, Pulsed Radiofrequency[Title/Abstract])) OR (Treatments, Pulsed Radiofrequency[Title/Abstract])) OR (Pulsed Radio Frequency Treatment[Title/Abstract]))
Cochrane library	#1 MeSH descriptor: [Neuralgia, Postherpetic] explode all trees #2 (Neuralgia, Postherpetic):ti,ab,kw OR (Postherpetic Neuralgia):ti,ab,kw #3 #1 OR #2 #4 MeSH descriptor: [Spinal Cord Stimulation] explode all trees #5 (Spinal Cord Stimulation):ti,ab,kw OR (Cord Stimulation, Spinal):ti,ab,kw OR (Stimulation, Spinal Cord):ti,ab,kw #6 #4 OR #5 #7 MeSH descriptor: [Pulsed Radiofrequency Treatment] explode all trees #8 (Pulsed Radiofrequency Treatment):ti,ab,kw OR (Pulsed Radiofrequency Treatments):ti,ab,kw OR (Radiofrequency Treatment, Pulsed):ti,ab,kw OR (Radiofrequency Treatments, Pulsed):ti,ab,kw OR (Treatment, Pulsed Radiofrequency):ti,ab,kw OR (Treatments, Pulsed Radiofrequency):ti,ab,kw OR (Pulsed Radio Frequency Treatment):ti,ab,kw #9 #7 OR #8 #10 #3 AND #6 AND #9
Web of science	((TS = (Neuralgia, Postherpetic)) OR TS = (Postherpetic Neuralgia)) AND (((TS = (Spinal Cord Stimulation)) OR TS = (Cord Stimulation, Spinal)) OR TS = (Stimulation, Spinal Cord)) AND (((((((TS = (Pulsed Radiofrequency Treatment)) OR TS = (Pulsed Radiofrequency Treatments)) OR TS = (Radiofrequency Treatment, Pulsed)) OR TS = (Radiofrequency Treatments, Pulsed)) OR TS = (Treatment, Pulsed Radiofrequency)) OR TS = (Treatments, Pulsed Radiofrequency)) OR TS = (Pulsed Radio Frequency Treatment))
Embase	#10. #3 AND #6 AND #9 #9. #7 OR #8 #8. ‘pulsed radiofrequency treatment’:ab,ti #7. ‘pulsed radiofrequency treatment’/exp #6. #4 OR #5 #5. ‘spinal stimulation’:ab,ti OR ‘spinal cord stimulation’:ab,ti #4. ‘spinal cord stimulation’/exp. #3. #1 OR #2 #2. ‘postherpetic neuralgia’:ab,ti OR ‘herpetic neuralgia’:ab,ti OR ‘neuralgia, postherpetic’:ab,ti OR ‘postherpetic pain’:ab,ti #1. ‘postherpetic neuralgia’/exp
CBM-disk	“带状疱疹后神经痛”[常用字段:智能] AND “脊髓电刺激”[常用字段:智能] AND “脉冲射频”[常用字段:智能] Translation: “Postherpetic Neuralgia” [Common field: intelligent] AND “Spinal Cord Stimulation” [Common field: intelligent] AND “Pulsed Radiofrequency” [Common field: intelligent]
CNKI	(篇关摘:带状疱疹后神经痛(模糊)) AND (篇关摘:脊髓电刺激(模糊)) AND (篇关摘:脉冲射频(模糊)) Translation: (Abstract: Postherpetic Neuralgia (Fuzzy)) AND (Abstract: Spinal Cord Stimulation (Fuzzy)) AND (Abstract: Pulsed Radiofrequency (Fuzzy))
Wanfang	题名或关键词:(带状疱疹后神经痛) AND 题名或关键词:(脊髓电刺激) AND 题名或关键词:(脉冲射频) Translation: Title or keywords: (Postherpetic Neuralgia) AND Title or keywords: (Spinal Cord Stimulation) AND Title or keywords: (Pulsed Radiofrequency)
VIP	((题名或关键词 = 带状疱疹后神经痛 AND 题名或关键词 = 脊髓电刺激) AND 题名或关键词 = 脉冲射频) Translation: ((Title or keywords = Postherpetic Neuralgia AND Title or keywords = Spinal Cord Stimulation) AND Title or keywords = Pulsed Radiofrequency)

### 2.2 Inclusion and exclusion criteria

Our inclusion criteria were as follows: (1) Patients: patients diagnosed with PHN; (2) Study type: Randomized Controlled Trail (RCT); (3) Intervention measures: stSCS group received stSCS treatment, and PRF group received PRF treatment; and (4) Outcome indicators: Visual Analogue Scale (VAS) or Numerical Rating Scale (NRS), Pittsburgh Sleep Quality Index (PSQI), Pain Rating Index Affective (PRI-A), Pain Rating Index Sensory (PRI-S), effective rate (pain score decreases by > 50%), complete remission rate (pain score decreases by > 75% or VAS score < 3), adverse events, and treatment costs.

The exclusion criteria were as follows: (1) the research design was unreasonable; (2) the full text could not be obtained; (3) the data were completely missing; (4) duplicate research; and (5) studies published in languages other than Chinese and English.

### 2.3 Study screening and data extraction

Two researchers separately screened the studies according to the inclusion and exclusion criteria established in advance. Any disagreements were resolved through discussion or arbitration by a third researcher. The retrieved studies were imported into EndNote X9 to remove duplicates. Titles, abstracts, and full texts were carefully reviewed to identify eligible studies. Data extraction was performed using Excel spreadsheets and included (authors, year, sample size, duration of PHN, target, available outcomes, and follow-up points).

### 2.4 Quality evaluation

The risk of bias assessment tool of the Cochrane Collaboration was used to evaluate the RCTs that met the inclusion criteria. The evaluations included the quality of the included trials, such as random sequence generation, allocation concealment, blinding of participants and personnel, blinding of outcome assessment, incomplete outcome data, selective reporting, and other sources of bias.

### 2.5 Statistical analysis

Review Manager 5.4 and Stata 18.0 were used for statistical analysis. When the outcome index was dichotomous data, relative risk (RR) and 95% confidence interval (CI) were used for quantitative analysis. When the outcome index was a continuous data, mean difference (MD) and 95% CI were used for quantitative analysis. If the indicators were measured in different ways, the standardized mean difference (SMD) and 95% CI were used for quantitative analysis. The Higgins *I^2^* and *Q* tests were used for heterogeneity analysis. If *I^2^* ≥ 50% or *Q* test < 0.1, the heterogeneity was obvious and the random-effect model was used for analysis. Otherwise, a fixed-effect model or random-effect model was used for the analysis. For results with obvious heterogeneity, subgroup analysis was used to determine the source of heterogeneity. Furthermore, we will explore some potential sources of heterogeneity. To assess the robustness of our findings, we conducted a series of sensitivity analyses by excluding individual studies one at a time and examining the impact on the overall results. The Egger’s test was performed to detect publication bias. Subgroup analysis, sensitivity analysis, and publication bias analysis might not be feasible or appropriate if the number of included studies was limited. Differences were considered statistically significant for *p* < 0.05.

## 3 Results

### 3.1 Literature search results

**Figure 1:**
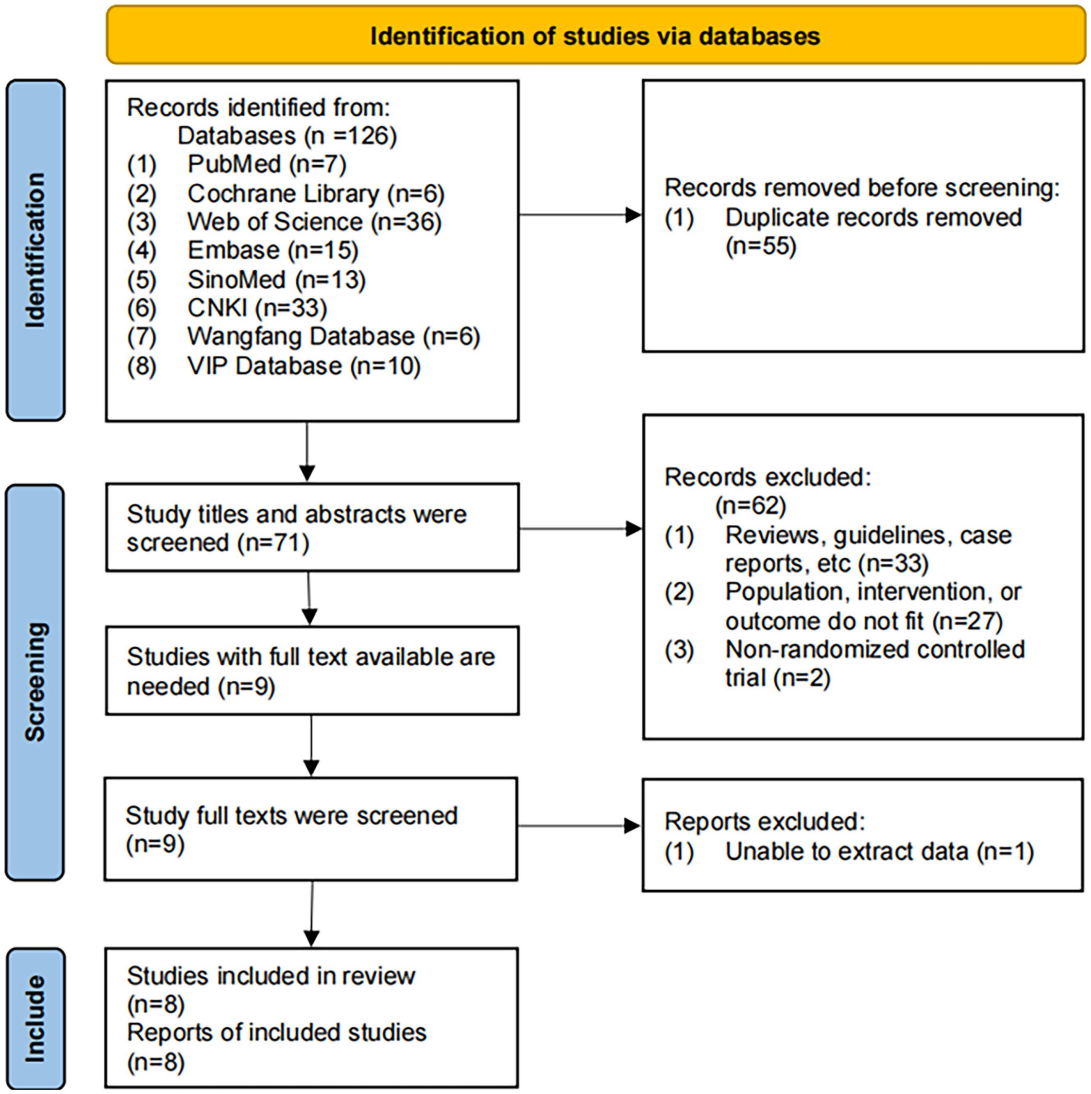
The PRISMA flow chart of literature screening.

A total of 126 studies were identified by searching eight databases, and 55 duplicate studies were excluded. A preliminary screening was performed by reading the titles and abstracts of the remaining studies, and 62 articles were excluded. After reading the remaining nine full texts, we conducted a second screen. One study was excluded because data could not be extracted. Finally, the remaining eight studies (31–38) were included in our study. A flowchart of the literature screening process is shown in Figure 1.

### 3.2 Basic characteristics of literature

**Figure 2:**
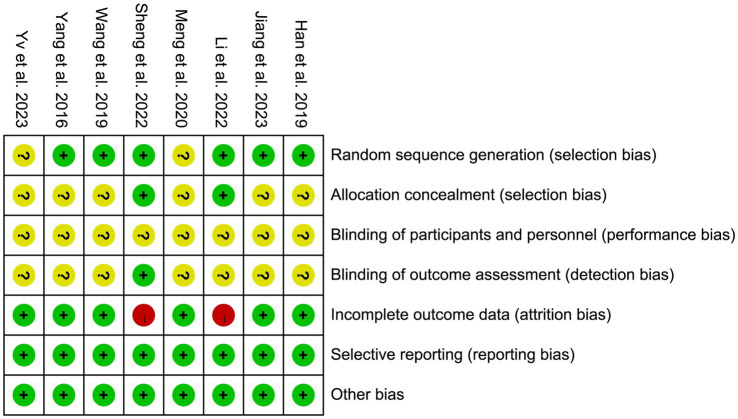
Risk of bias summary.

We included two studies in English (36, 37) and six in Chinese (31–35, 38). A total of 479 patients were included. The basic characteristics of the included studies are presented in Table 2. The results of study quality evaluation are shown in Figure 2.

**Table 2:** Characteristics of the included studies.

Authors	Year	Sample size (S/P)	Duration of PHN	Target	Intervention	Available outcomes	Follow-up points
Sheng et al. (36)	2022	30/40	S: 2.94 M P: 3.19 M	S: Dorsal column P: DRG	S: 1–3 V, 20–80 Hz, 210–450 μs, 14 days P: 40–100 V, 2 Hz, 20 ms, 42°C, 600 s, 2 sessions	Effective rate Adverse events	1 D, 1 W, 1 M, 3 M, 6 M, 12 M
Li et al. (37)	2022	22/22	S: 55 D P: 47.5 D	S: Dorsal column P: DRG	S: 2 V, 40 Hz, 210 μs, 7 days P: 2 Hz, 42°C, 120 s, 2 sessions	Effective rate Complete remission rate Adverse events	1 D, 1 W, 1 M, 3 M, 6 M
Yv et al. (35)	2023	20/20	S: 6.45 M P: 6.54 M	S: Dorsal column P: Peripheral nervous	S: 0.5–5 mA, 50 Hz, 180–550 μs P: 99 V, 2 Hz, 20 ms, 42°C, 360 s, 2 sessions	NRS PSQI Complete remission rate Adverse events	1 M
Jiang et al. (31)	2023	67/68	S: 26.84 M P: 26.73 M	S: Dorsal column P: DRG	S: 0.8–3.2 V, 60–80 Hz, 60–80 μs, 7 days P: 72 V, 2 Hz, 20 ms, 42°C, 360 s, 14 sessions	VAS PSQI Complete remission rate Adverse events	1 W, 1 M, 3 M
Meng et al. (32)	2020	30/30	S: 45.7 D P: 46.3 D	S: Dorsal column P: DRG	S: 1–3 V, 50–80 Hz, 120–180 μs, 7 days P: 2 Hz, 20 ms, 42°C, 120 s, 2 sessions	VAS PSQI	1 M, 3 M, 6 M
Yang et al. (34)	2016	20/20	S: 6.8 M P: 6.9 M	S: Dorsal column P: DRG	S: 0.8–3.2 V, 60–180 Hz, 20–200 μs, 10–14 days P: 2 Hz, 20 ms, 42°C, 480 s	VAS PRI-A PRI-S Adverse events	1 D, 1 W, 1 M, 2 M, 3 M
Wang et al. (33)	2019	20/20	S: 3.2 M P: 3.1 M	S: Dorsal column P: DRG	S: 0.8–3.2 V, 60–80 Hz, 60–80 μs, 10 days P: 2 Hz, 20 ms, 42°C, 120 s, 2 sessions	Adverse events Treatment costs	3 D, 10 D, 1 M, 2 M, 3 M
Han et al. (38)	2019	25/25	S: 26.78 M P: 26.53 M	S: Dorsal column P: DRG	S: 10–14 days P: Not reported	VAS PRI-A PRI-S PSQI Adverse events	10 D, 1 M, 2 M

VAS, Visual Analogue Scale; NRS, Numerical Rating Scale; PRI-A, Pain Rating Index Affective; PRI-S, Pain Rating Index Sensory; PSQI, Pittsburgh Sleep Quality Index.

S, short-term Spinal Cord Stimulation; P, Pulse Radio Frequency.

DRG, Dorsal Root Ganglion.

D, Day; W, Week, M, Month.

### 3.3 Meta-analysis results

#### 3.3.1 Pain intensity

**Figure 3:**
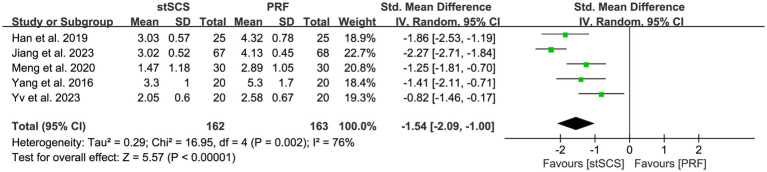
Forest plot of pain intensity at the last follow-up.

**Figure 4:**
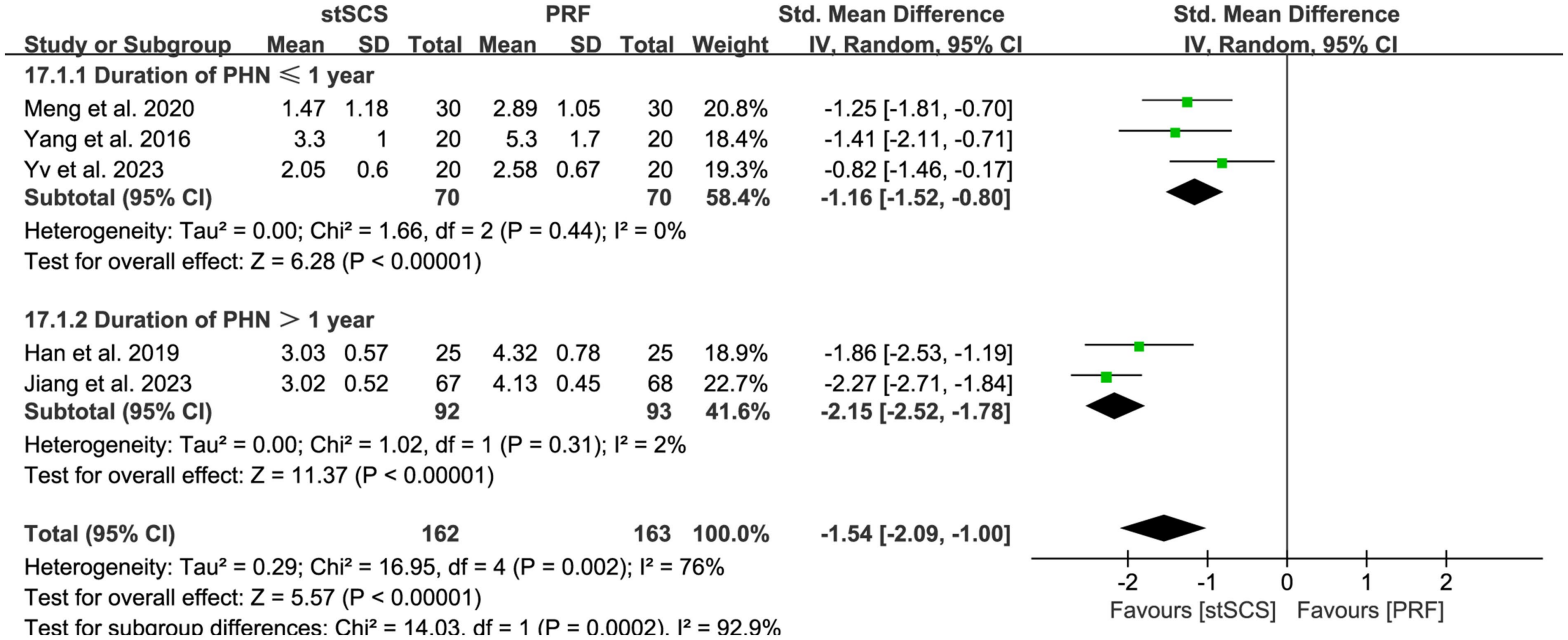
Forest plots of pain intensity for different duration.

Data related to pain intensity that was evaluated using either the VAS or NRS were extracted from five studies (31, 32, 34, 35, 38). The heterogeneity between studies was obvious (*I*^2^ = 76%, *p* = 0.002), prompting the use of a random-effects model for analysis. Compared with PRF, the pain intensity relief in the stSCS group was more significant (SMD = −1.54, 95% CI: −2.09 to −1.00, *p* < 0.01) (Figure 3). Furthermore, subgroup analysis was performed based on the duration of the disease, and the results showed that stSCS was more effective than PRF in reducing the degree of pain in patients with a disease duration of ≤ 1 year or > 1 year (Figure 4).

Quantitative analyses were performed at each follow-up visit. The results showed that the stSCS group had better pain relief in the first week (MD = −1.24, 95% CI: −1.70 to −0.79, *p* < 0.01), first month (SMD = −1.70, 95% CI: −2.36 to −1.04, *p* < 0.01), second month (MD = −1.67, 95% CI: −2.55 to −0.79, *p* < 0.01), and third month (MD = −1.35, 95% CI: −1.78 to −0.92, *p* < 0.01) after treatment (Table 3).

**Table 3:** Meta-analysis of outcomes at each time point after treatment.

Outcome	Follow-up time	Number of studies	Heterogeneity		Model	Results	
			*p*	*I^2^* (%)		MD/SMD (95% CI)	*p*-value
Pain intensity	1W	2	0.16	50	Random	−1.24 (−1.70 to −0.79)	< 0.01
	1M	5	< 0.01	83	Random	−1.70 (−2.36 to −1.04)	< 0.01
	2M	2	0.04	75	Random	−1.67 (−2.55 to −0.79)	< 0.01
	3M	3	0.10	57	Random	−1.35 (−1.78 to −0.92)	< 0.01
PRI-A	1M	2	< 0.01	91	Random	−6.15 (−8.48 to −3.82)	< 0.01
	2M	2	0.003	89	Random	−5.76 (−7.84 to −3.68)	< 0.01
PRI-S	1M	2	< 0.01	94	Random	−2.85 (−4.50 to −1.20)	< 0.01
	2M	2	< 0.01	93	Random	−2.55 (−4.02 to −1.08)	< 0.01
PSQI	1M	4	< 0.01	96	Random	−1.82 (−4.00 to 0.36)	0.10
Effective rate	1D	2	0.11	62	Random	1.28 (0.84 to 1.94)	0.25
	1W	2	0.007	86	Random	1.32 (0.68 to 2.54)	0.41
	1M	2	0.01	84	Random	1.29 (0.68 to 2.43)	0.43
	3M	2	0.03	79	Random	1.99 (1.08 to 3.68)	0.03
	6M	2	0.80	0	Fixed	1.57 (1.20 to 2.06)	0.001
Complete remission rate	1M	2	0.03	79	Random	2.00 (0.63 to 6.29)	0.24
	3M	2	< 0.01	95	Random	2.92 (0.22 to 38.97)	0.42

PRI-A, Pain Rating Index Affective; PRI-S, Pain Rating Index Sensory; PSQI, Pittsburgh Sleep Quality Index.

MD, Mean Difference; SMD, Standardized Mean Difference.

D, Day; W, Week; M, Month.

#### 3.3.2 Pain grading index

**Figure 5:**
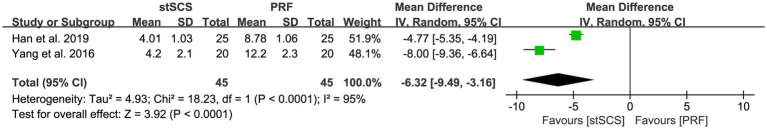
Forest plot of PRI-A at the last follow-up.

**Figure 6:**
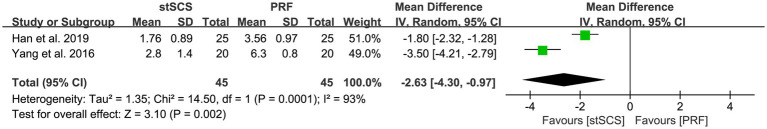
Forest plot of PRI-S at the last follow-up.

Two studies (34, 38) reported the PRI-A/S scores. For the PRI-A score, significant heterogeneity was observed between the studies (*I*^2^ = 95%, *p* < 0.01), necessitating the use of a random-effects model. The results showed that, compared with PRF therapy, the PRI-A score decreased more significantly after stSCS treatment (MD = −6.32, 95% CI: −9.49 to −3.16, *p* < 0.01) (Figure 5). Similarly, for the PRI-S score, substantial heterogeneity was also noted (*I*^2^ = 93%, *p* < 0.01), and a random-effects model was used. The results showed that, compared with PRF therapy, the PRI-S score decreased more significantly after stSCS treatment for PHN (MD = −2.63, 95% CI: −4.30 to −0.97, *p* = 0.002) (Figure 6).

The reduction in the PRI-A and PRI-S scores was more significant in the stSCS group in both the first and second months after treatment (PRI-A: the first month [MD = −6.15, 95% CI: −8.48 to −3.82, *p* < 0.01], the second month [MD = −5.76, 95% CI: −7.84 to −3.68, *p* < 0.01]. PRI-S: the first month [MD = −2.85, 95% CI: −4.50 to −1.20, *p* < 0.01], the second month [MD = −2.55, 95% CI: −4.02 to −1.08, *p* < 0.01]) (Table 3).

#### 3.3.3 Sleep quality

**Figure 7:**
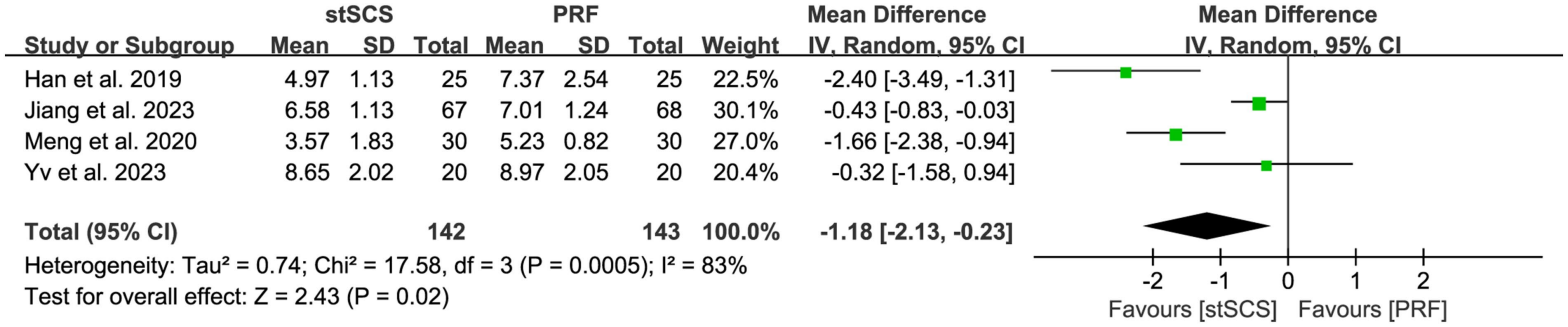
Forest plot of PSQI at the last follow-up.

Four studies (31, 32, 35, 38) used PSQI to assess sleep quality. The heterogeneity between studies was obvious (*I*^2^ = 83%, *p* < 0.01), and a random-effects model was used. The results showed that compared with PRF therapy, patients after stSCS treatment for PHN had lower PSQI scores (MD = −1.18, 95% CI: −2.13 to −0.23, *p* = 0.02) (Figure 7).

No significant differences were observed in the first month after treatment (MD = −1.82, 95% CI: −4.00 to 0.36, *p* = 0.10) (Table 3). In the third month after treatment, excessive heterogeneity was observed between two studies (31, 32) (*I*^2^ = 97%, *p* < 0.01), precluding quantitative analysis. Both the studies reported improved sleep quality in the stSCS group.

#### 3.3.4 Effective rate and complete remission rate

**Figure 8:**
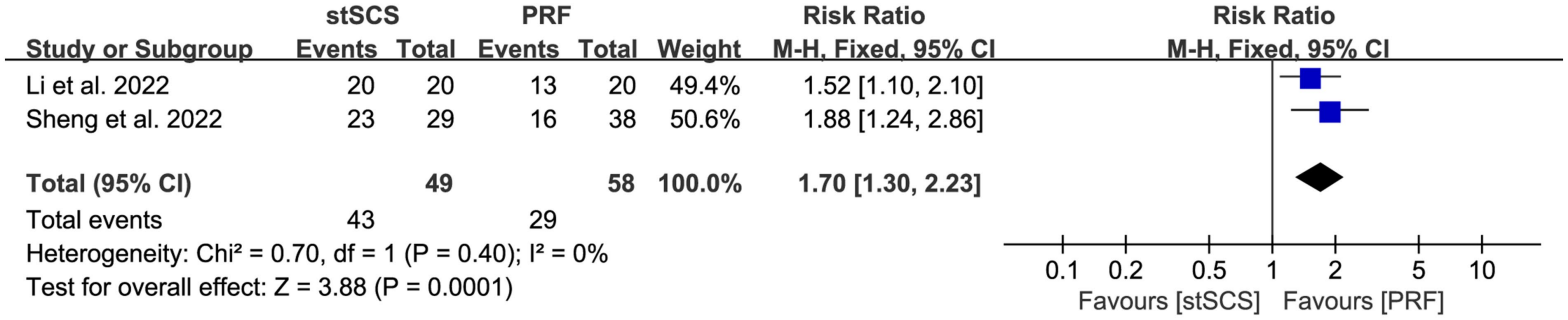
Forest plot of effective rate at the last follow-up.

Two studies (36, 37) determined the effective rate. No significant heterogeneity was observed among the studies (*I*^2^ = 0%, *p* = 0.40), and a fixed-effects model was used. The results showed that the stSCS group had a higher effective rate (RR = 1.70, 95% CI: 1.30 to 2.23, *p* < 0.01) (Figure 8).

**Figure 9:**
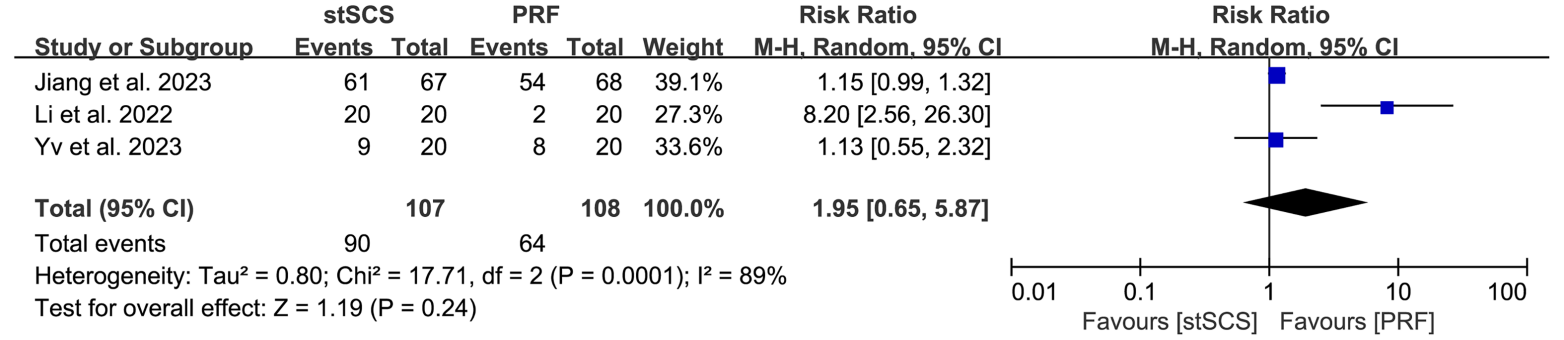
Forest plot of complete remission rate at the last follow-up.

Three studies (31, 35, 37) reported the complete remission rate. The heterogeneity between studies was obvious (*I*^2^ = 89%, *p* < 0.01), and a random-effects model was used. There was no statistically significant difference in the complete remission rate between the two methods for treating PHN (RR = 1.95, 95% CI: 0.65 to 5.87, *p* = 0.24) (Figure 9).

During the first day (RR = 1.28, 95% CI: 0.84 to 1.94, *p* = 0.25), first week (RR = 1.32, 95% CI: 0.68 to 2.54, *p* = 0.41), and first month (RR = 1.29, 95% CI: 0.68 to 2.43, *p* = 0.43) after treatment, no statistically significant differences were observed in the effective rate between the stSCS and PRF groups. However, in the third (RR = 1.99, 95% CI: 1.08 to 3.68, *p* = 0.03) and sixth (RR = 1.57, 95% CI: 1.20 to 2.06, *p* = 0.001) months after treatment, the stSCS group had a higher effective rate. No significant differences were observed in complete remission rate between the stSCS and PRF groups in the first (RR = 2.00, 95% CI: 0.63 to 6.29, *p* = 0.24) and third (RR = 2.92, 95% CI: 0.22 to 38.97, *p* = 0.42) months after treatment (Table 3).

#### 3.3.5 Adverse events

**Figure 10:**
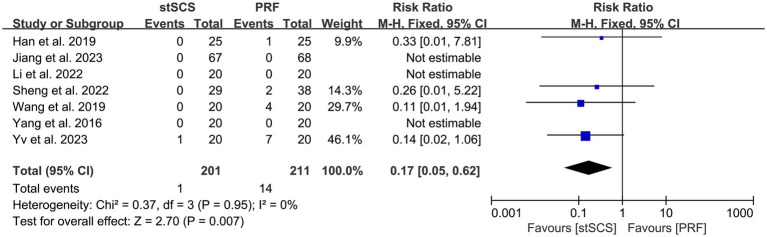
Forest plot of complication.

Seven studies (31, 33–38) observed the occurrence of postoperative complications in patients, with four (33, 35, 36, 38) of them reporting complications in patients. The heterogeneity between studies was not obvious (*I*^2^ = 0%, *p* = 0.95), and a fixed-effects model was used. The results showed that the incidence of complications after stSCS was lower than that after PRF (RR = 0.17, 95% CI: 0.05 to 0.62, *p* = 0.007) (Figure 10).

#### 3.3.6 Treatment costs

Only one study (33) recorded costs related to patient treatment, precluding the possibility of conducting a statistical analysis. This study showed that PRF treatment is less costly than stSCS treatment.

#### 3.3.7 Heterogeneity, sensitivity, and publication bias

Given the substantial heterogeneity observed across study outcomes, we conducted an analysis of the potential sources of heterogeneity. We believe that the heterogeneity in outcomes may stem from several factors, including differences in the duration of patients’ pain, discrepancies in the selected intervention protocols (e.g., the choice of intervention segments, PRF parameters such as voltage and duration, number of PRF treatment sessions, PRF target sites such as peripheral nerves or dorsal root ganglia, stSCS parameters, and duration of stSCS treatment), inconsistency in the timing of the final follow-up, and the predominance of subjective outcome measures based on rating scales. Sensitivity analyses were conducted by excluding individual studies. When we excluded studies at the last follow-up of the PSQI individually, the results of the meta-analysis changed, indicating that the results were unstable. Sensitivity analyses of the remaining results showed that the overall effect size in each group did not change significantly, indicating that the results of the meta-analysis were relatively stable. Egger’s test was conducted for all outcomes that met the criteria, and no evidence of publication bias was observed. The Egger scores are presented in Table 4.

**Table 4:** Publication bias.

Test	Pain intensity	Sleep quality	complete remission rate	Adverse events
Egger’s test	0.193	0.392	0.484	0.408

## 4 Discussion

As a developmental outcome of refractory HZ, PHN has a significant impact on the quality of life of patients. Conventional drug treatment is not effective enough, and providing adequate analgesic effect to patients is difficult and is sometimes accompanied by certain side effects (39, 40). Both physical therapy and nerve block (41) have certain curative effects; however, maintaining pain relief over time remains difficult (42). In recent years, interventional therapy has gradually been accepted by patients due to its advantages of minimal invasion and good efficacy. Both stSCS (43) and PRF (44) are neuromodulation techniques that are increasingly used for pain treatment (45).

Although previous studies have explored the analgesic effects of stSCS and PRF, several limitations exist in the available literature. Therefore, we conducted this meta-analysis to provide more reliable and broadly applicable conclusions. We found that stSCS generally provides better therapeutic outcomes than PRF, with this advantage being maintained over the long term. Subgroup analysis of PHN with different durations (≤1 year or >1 year) was also conducted in this study, and the results were similar. Furthermore, through a quantitative analysis of studies with different follow-up durations, we observed that stSCS may be more meaningful than PRF in terms of maintaining therapeutic effects. Possible reasons for this are discussed below.

SCS includes a variety of modalities such as stSCS, permanent conventional SCS, high-frequency SCS, burst SCS, and dorsal root ganglion stimulation (12). Foreign countries tend to use permanent electrode implantation for mode selection, whereas China tends to use stSCS. The stimulation time of stSCS is 1–2 weeks, and does not require permanent electrode implantation. stSCS can provide satisfactory pain relief for patients at a low cost and is convenient. During SCS, the physician implants electrodes into the epidural space to stimulate the spinal nerves using a pulsed current. The current may act on Aβ fibers. Subsequent afferents from nerve fibers stimulate inhibitory interneurons to control the degree of pain and improve the quality of life of patients (8, 46). Additionally, SCS can relieve pain by promoting the release of *γ*-aminobutyric acid (47, 48), reducing the release of inflammatory mediators, and improving blood circulation (49). Analysis of the brain function of patients undergoing stSCS during the stimulation period by functional magnetic resonance imaging showed that stSCS could cause changes in the dynamic low-frequency amplitude of patients with PHN (43) and induce changes in regional homogeneity and degree centrality in patients (50), which may alter brain function to relieve pain, sleep, and mood symptoms. Sheng L et al. reported that the effective rate of pain control could be maintained at 79.3% after 12 months of stSCS treatment (36). Yanamoto et al. showed that stSCS had an effective rate of 63.6% at 6 months after PHN treatment (51). Dong et al. reported the efficacy of stSCS treatment in 46 patients with different courses of HZ-associated neuralgia and showed that stSCS could significantly improve pain intensity and reduce the use of analgesics, and these effects could be maintained for at least 12 months. The results of the comparison between the two groups showed that the duration of the disease had no effect on the treatment efficacy (13). However, large-sample prospective studies that can confirm the long-term effectiveness of stSCS are lacking.

The effectiveness and safety of PRF as an interventional treatment for PHN have been verified in clinical practice. PRF targets the nerve root or peripheral nerves. By generating a pulsed current, a magnetic field is formed at the needle tip to regulate nerve function. The mechanism of action of PRF involves regulating the expression and function of ion channels and the release of transmitters and inflammatory mediators. Currently, the optimal treatment mode for PRF has not been definitively determined, and different targets and parameters can produce different therapeutic effects. Huang et al. reported that PRF stimulation of the dorsal root ganglia reduced pain intensity much more than stimulation of peripheral nerves, which may be because the dorsal root ganglia are the main target of viral action (52). Wan et al. compared the therapeutic effects of standard voltage and standard duration with high-voltage and long-term PRF. Their results showed that high-voltage and long-term PRF can better relieve pain and reduce the use of analgesic drugs (53). Therefore, The different treatment modes of PRF in the literature included in this study may be the source of heterogeneity among studies. As the included study modes were not uniform or missing, further analysis of the different modes was not conducted.

Based on the above analysis, stSCS is superior to PRF in the treatment of patients with PHN in terms of pain control and sleep improvement, owing to its different mechanisms of action.

In terms of safety, Li et al. reported 44 patients with PHN were treated with stSCS and PRF without surgery-related complications (29). However, owing to the small sample size, the real safety situation could not be reflected. Sheng et al. also reported that stSCS is safer than PRF (36). Combined with clinical operational process analysis, stSCS may be a safer treatment method. First, during PRF treatment, the puncture site is rich in blood vessels and nerves and is difficult to locate in real-time. Second, during PRF treatment, multiple segments may need to be punctured because of the wide distribution of pain. The sample sizes of the studies reported to date are small. As the stSCS puncture involves the central nervous system, the occurrence of infection has serious consequences. Therefore, studies with large sample sizes are urgently needed to determine the complication rates of stSCS to provide a reference for clinical practice. In addition, the incidence of headache after dural puncture (54), a common clinical complication, has not been reported in studies on stSCS.

Second, the PQSI scores of patients after stSCS treatment were lower, indicating better sleep quality after stSCS treatment. This may be related to the mechanism by which stSCS changes brain function; therefore, stSCS can reduce PSQI scores to a greater extent.

Third, the efficacy rate of stSCS was higher than that of PRF; however, there was no significant difference in the complete response rates between the two therapies. The results also showed that there were no between-group differences in sleep quality or response rate in the early stage after treatment; however, there were significant between-group differences in the middle and late stages after treatment. Consistent with the results of other studies, stSCS was better than PRF in multi-dimensional evaluation (14). stSCS may be more durable than PRF in maintaining the therapeutic effect.

Fourth, in terms of clinical economics, Wang et al. compared the total cost of the two treatment methods (33). They found that the total hospitalization cost of stSCS was higher than that of PRF.

The limitations of this study are as follows: (1) the baseline conditions of patients in each study were slightly different; (2) there is no unified standard for the treatment modes of stSCS and PRF in PHN treatment; (3) whether other treatments are combined between studies is different; (4) the follow-up time after treatment between studies is different; and (5) most of the experimental outcome indicators are clinical scales, which are highly subjective. These factors may have impacted the study outcomes and introduced considerable heterogeneity into the quantitative analysis. Moreover, the follow-up time of the study was short, and long-term follow-up results are needed for further analysis. In addition, all experiments included in this study were conducted in China and were single-center experiments. Finally, the sample size of these trials and the number of articles included for individual outcomes were small. Therefore, the outcomes may have been unstable. Therefore, more high-quality studies are required to confirm these conclusions.

## 5 Conclusion

In conclusion, our results showed that stSCS can improve pain intensity and sleep quality to a greater extent than PRF in patients with PHN. Furthermore, stSCS exhibits higher effective rate and safety. However, stSCS is more expensive. The choice of the treatment method requires a comprehensive consideration of the advantages and disadvantages of the two methods.

## Data Availability

The original contributions presented in the study are included in the article/supplementary material, further inquiries can be directed to the corresponding authors.

## Footnotes

^1^ https://www.crd.york.ac.uk/PROSPERO/
